# Seasonal Abundance of Aphids and Aphidophagous Insects in Pecan

**DOI:** 10.3390/insects3041257

**Published:** 2012-12-05

**Authors:** James D. Dutcher, Haider Karar, Ghulam Abbas

**Affiliations:** 1 Entomology Department, University of Georgia, 2360 Rainwater Rd., Tifton, GA 31793, USA; 2 Entomological Research Sub-Station, Old Shujabad Road, Multan 60000, Pakistan; E-Mail: haider853@gmail.com; 3 Pest Warning & Quality Control of Pesticides, Punjab, Khanewal 58150, Pakistan; E-Mail: drabbas18@gmail.com

**Keywords:** shade tree, biological aphid and mite control, insect and mite predators

## Abstract

Seasonal occurrence of aphids and aphidophagous insects was monitored for six years (2006–2011) from full leaf expansion in May to leaf fall in October in “Desirable” variety pecan trees that were not treated with insecticides. Aphid outbreaks occurred two times per season, once in the spring and again in the late summer. Yellow pecan and blackmargined aphids exceeded the recommended treatment thresholds one time and black pecan aphids exceeded the recommended treatment levels three times over the six seasons. Increases in aphidophagous insect abundance coincided with aphid outbreaks in five of the six seasons. Among aphidophagous insects *Harmonia axyridis* and *Olla v-nigrum* were frequently collected in both the tree canopy and at the ground level, whereas, *Coccinella septempunctata*, *Hippodamia convergens* were rarely found in the tree canopy and commonly found at the ground level. Green lacewing abundance was higher in the ground level than in the tree canopy. Brown lacewings were more abundant in the tree canopy than at the ground level. Dolichopodid and syrphid fly abundance, at the ground level increased during peak aphid abundance in the tree canopy. Application of an aqueous solution of fermenting molasses to the pecan foliage during an aphid outbreak significantly increased the abundance of ladybeetles and lacewings and significantly reduced the abundance of yellow pecan, blackmargined and black pecan aphids.

## 1. Introduction

Pecan, *Carya illinonensis* (Wangenh.) K. Koch, is an important source of nuts, lumber and shade, especially within its native range in the mid-southern United States. Pecan has many potential insect and mite pests that detract from its value in urban plantings in the southeastern U.S. Insects and/or mites feed upon and cause injury to every major system of the tree [1]. Integration of biological and chemical controls has produced management schemes to quell insect outbreaks quickly and with high effectiveness in the commercial pecan orchards. However, only the biological controls are recommended for urban pecan trees or organic pecan orchards [2]. The trees typically are large, 10–22 m in height and covering an area of 0.04 ha. The trees are difficult to treat with pesticides and homeowners rely on natural enemies for pest control [3]. Although pecan trees provide shade from May to October and pecan nuts in November and December, a major drawback to pecan as a yard tree is the degradation of the foliage by three species of aphids [4]—*Monellia caryella* (Davis), *Monelliopsis pecanis* Bissell and *Melanocallis caryaefoliae* (Granovsky) (Hemiptera: Aphididae)—that often occur as outbreaks during the spring and late summer. The aphids produce honeydew that rains down from the infested foliage onto lower leaves and any structures or vehicles below the tree crown. Sooty mold then grows on the accumulated honeydew. Severe infestations lead to defoliation as early as late August. In commercial plantings these outbreaks often require control with aphidicides, applied as sprays to the foliage. One approach to timing the spray for aphid control is based on “action thresholds” where aphid populations are treated when the combined count for yellow pecan and blackmargined aphids exceeds 20 aphids per leaf or the count for black pecan aphids exceeds 1 aphid per leaf [2,5]. Yellow pecan and blackmargined aphids are evenly distributed in the tree [6], whereas, black pecan aphids initially colonize the foliage of the lower branches of the interior of the tree canopy and then spread to the foliage of periphery and upper branches [4]. Fortunately, a complex of natural and introduced aphidophagous insects prey on the aphids throughout the season [7] and biological control with these predators is an effective and sustainable method for maintaining relatively low aphid populations and preventing outbreaks and subsequent honeydew accumulation and early defoliation. Field bioassays determined that the introduced aphidophagous beetle, *Harmonia axyridis* (Pallas) [Coleoptera: Coccinellidae] effectively controlled all three species of foliar pecan aphids in a four week period [8,9]. Chemical control of pecan aphids is highly effective in the short term but is fraught with problems with pest resurgence and replacement [10,11]. Host plant resistance may also be important, in the future, as a biological control of aphids on pecan. Cultivars vary considerably in susceptibility to aphids [12], however, the cultivars that are commonly grown—“Schley”, “Stuart”, and “Desirable”—are highly susceptible to one or more of the foliar-feeding aphid species. Among 23 cultivars observed in a recent study [13] five cultivars had significantly more aphids season-long than the three cultivars with the lowest abundance of aphids—“Amling”, “Lager” and “Gafford”. All other cultivars had intermediate abundance of aphids. The field biology of the aphidophagous insects is not well-known in pecan orchards. In the investigations, reported herein, our objectives were to determine how often aphids exceeded the action thresholds in pecan trees not treated with insecticides and measure the occurrence of major groups of aphidophagous insects in the orchard. We also compared the season-long abundance of aphidophagous insects in the canopy of the pecan trees and at the ground level and compared the abundance of the aphid populations in the pecan trees to the abundance of the predators. We also tested a “food spray” as an enhancement method for aphidophagous insects. In field trials measuring the effectiveness of food sprays for conservation of biological control agents over a 40 year period indicated an increase in natural enemies in 87% of the trials and a decrease in the pest population in 47% of the trials [14]. We were encouraged by the success of this method in pecan [15] and apple [16] and tested the effect of a food spray of molasses and baker’s yeast on the abundance of predators in the pecan tree canopy in an attempt to find an effective method for attracting the aphidophagous insects to the pest aphid infestations.

## 2. Results and Discussion

### 2.1. Seasonal Occurrence of Aphids and Aphidophagous Insects

**Figure 1:**
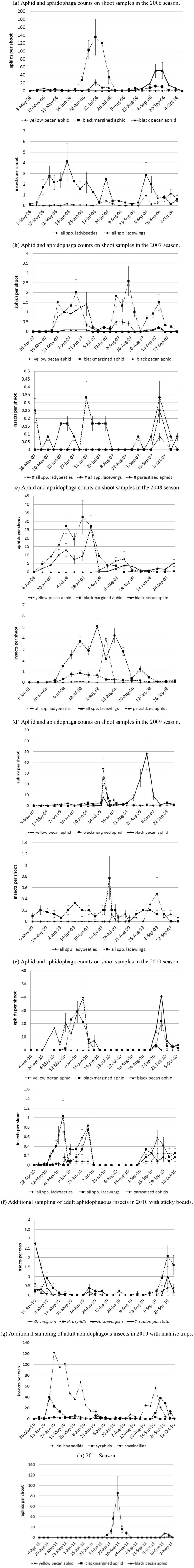
Seasonal occurrences of three species of aphids and associated aphidophagous insects on pecan foliage based on weekly counts over six seasons are as a series of figures indicating the mean abundance with errors bars representing the +/− standard error of the mean.

The monitoring of shoot samples for aphids and aphidophaga from 2006–2011 indicated that the three species of aphids increased rapidly during an outbreak and that peak abundance is not sustained for more than two weeks before the population crashes. The natural rates of increase for the three aphid species [17] are sufficient to achieve these rapid increases but the rapid declines could be caused by debilitation of vascular system in the pecan leaf [18], natural enemies [19,20], high temperature [21], rainfall [22] or some combination of factors [15]. The overall abundance of aphids and the periods of significant outbreaks differ considerably from season to season in pecan trees under the uniform and consistent cultural practices applied to our experimental site. In 2006, yellow pecan aphids remained at low abundance for the season. Blackmargined aphids increased in mid-summer from 22 to 103 aphids per shoot in 1 w, remained above 100 aphids per shoot for 2 w and then declined from 120 to 35 aphids per shoot in 1 w. Black pecan aphids increase in the fall from 19 to 53 aphids per shoot in one week. Lacewing activity increased before the blackmargined aphid peak and at the same time as the black pecan aphid peak, whereas, ladybeetle abundance increased 1 w. after the blackmargined aphid peak and at the same time as the black pecan aphid peak (Figure 1a). In 2007, populations and the three aphids and ladybeetles, lacewings and parasitized aphids were low for the season (Figure 1b). In 2008, the yellow pecan and blackmargined aphids occurred in a broad (6 w) period at moderate abundance relative to 2006. Black pecan aphids had a peak of abundance in the late summer and the ladybeetle abundance was low for the season. Parasitized aphids were more abundant than lacewings and the peak activity was a broad (8 w) period in the mid-summer (Figure 1c). In 2009, yellow pecan, blackmargined, and black pecan aphids increased in abundance for 1 w in mid-July and then quickly declined. A second outbreak of black pecan aphids occurred from late August to early September and reached a peak abundance of 48 aphids per shoot that lead to early defoliation of the trees in early October. Ladybeetles were low in abundance until 1 w after the start of the black pecan aphid outbreak when a broad (5 w) period of high abundance occurred. Lacewings were found and parasitized aphids were not found, season-long (Figure 1d). In 2010, yellow pecan and blackmargined aphids had a peak of activity in the spring that extended into the early summer and crashed to a zero and remained at zero throughout July and August. A mixed population outbreak of yellow pecan, blackmargined and black pecan aphids began in early September and extended in early October. Both peaks of aphid activity were accompanied by increases in lacewings and parasitized aphids and to a lesser extent, ladybeetles. Additional sampling of adult aphidophagous insects in 2010 with sticky boards (Figure 1f) and malaise traps at the ground level (Figure 1g), indicated that 4 species of ladybeetles (Coleoptera: Coccinellidae)—*Olla v-nigrum* (Mulsant), *Harmonia axyridis* (Pallas) *Hippodamia convergens* (Guérin-Méneville) and *Coccinella septempunctata* (L.)—and 2 groups of aphidophagaous flies—dolichopodid and syrphid flies—were present in the orchard at low levels from the onset of sampling in mid-April and throughout the season with peaks of trap catch corresponding to the periods of peak aphid abundance. The two malaise traps captured up to 122 adult dolochopodid flies per trap on 27 April 2010 and these were the most abundant aphidophagous insects collected in the traps (Figure 1e). These results indicate residual populations of important aphidophagous insects of the pecan aphids inhabit the ground level of the orchard throughout the season. In 2011, yellow pecan aphids were not found in the samples. Blackmargined aphids occurred in a mid-summer (late July to mid-August) peak of abundance that crashed after one week. Black pecan aphids occurred only very late in the fall (Figure 1h). Aphidophagous insects were only incidentally found in the foliage samples for the entire season. Even though the shoot sampling results indicated that lacewing and parasitized aphid were present over long periods before and after the aphid outbreak periods, these counts are not indicative of the number of living lacewings or parasitoids at the time of the sample. Hatched lacewing eggs and empty parasitized aphid mummies persist on the foliage from one sample date to the next. Ladybeetle counts, however, in the shoot samples incl. only live eggs, larvae, pupae or adults and are indicative of the number of insects preying on the aphids at the time of the sample.

**Figure 2:**
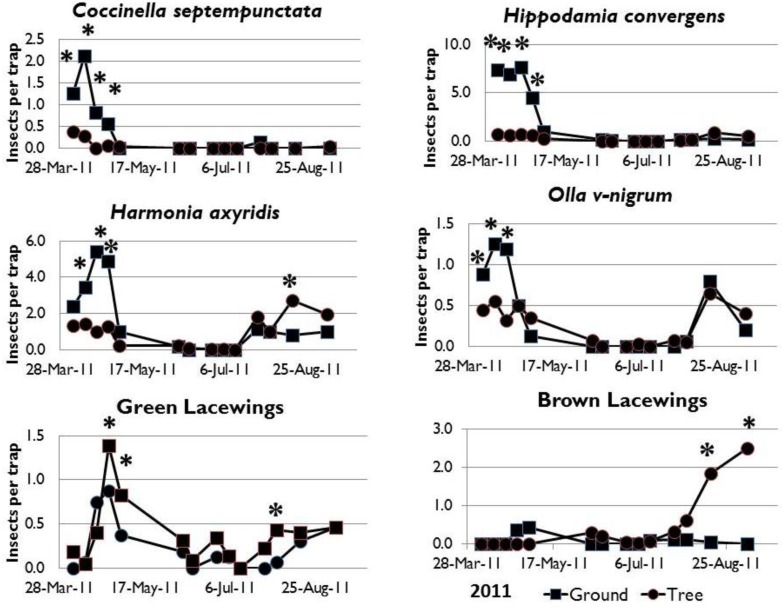
Comparison of sticky board trap catch of aphidophagous insects at the ground level (square marker) and in the pecan tree canopy (circle marker) in 2011. An asterisk above the data line indicates that on the indicated sample date the mean trap catch of the insect on the ground was significantly different from the mean trap catch in the tree canopy (*p* < 0.05, one-way ANOVA, n = 16 traps per level).

The results of the sticky board catch in 2011 yielded a good comparison of the aphidophagous insects in the tree canopy to the catch of traps at the ground level indicating two peaks of activity in the early spring (late March to early May) and the mid-summer (late July to late-August) activity period (Figure 2). There were significantly more adult *C. septempunctata* and *H. convergens* collected at the ground level than in the tree canopy for the entire season. *Harmonia axyridis*, adult catch was significantly higher at the ground level in the spring and significantly higher in the tree canopy in the mid-summer. *Olla v-nigrum* adult catch was significantly higher at the ground level in the spring and similar at the two levels in the mid-summer. Green lacewing adult catch was similar at both levels over the entire trapping period. Brown lacewing adult catch indicated a mid-summer peak of abundance in the tree canopy and incidental occurrence at the ground level and in the spring in the tree canopy. 

### 2.2. Effect of “Food Spray” on Abundance of Aphids and Aphidophagous Insects

**Figure 3:**
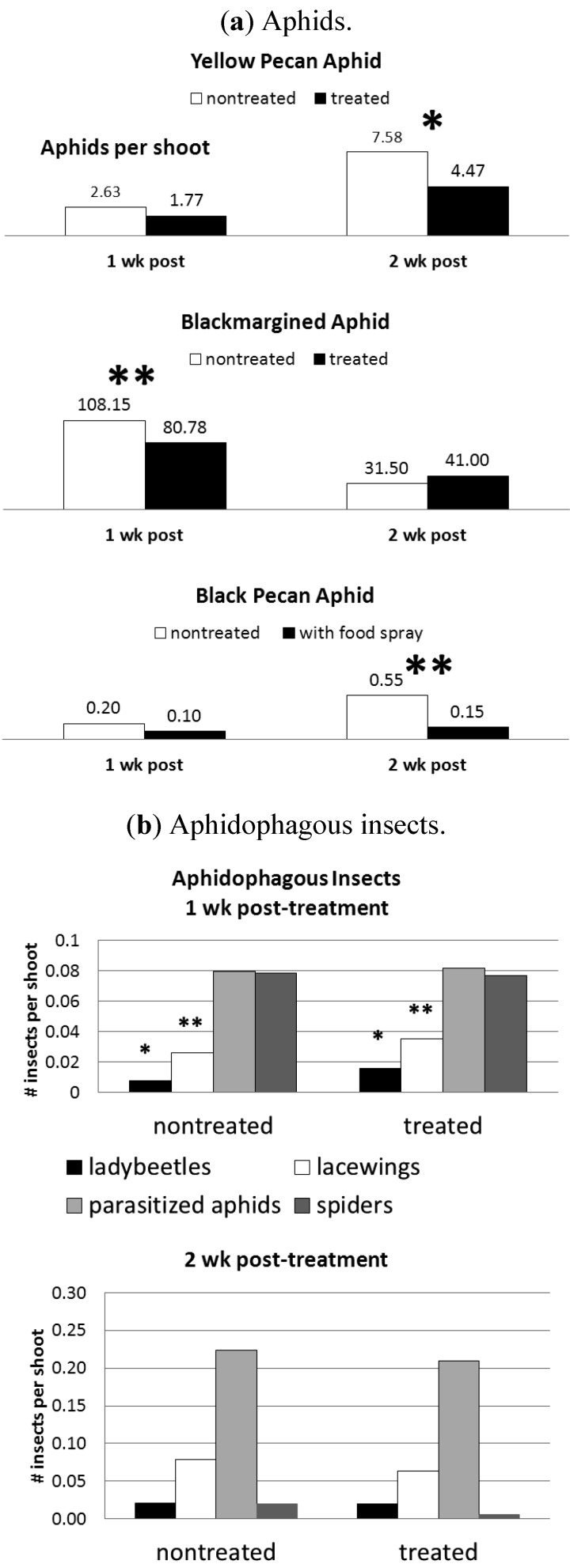
Comparison of aphid and aphidophagous insect abundance on pecan foliage in nontreated trees and trees treated with an aqueous solution of molasses and baker’s yeast, on sampling dates of 1 and 2 w after treatment. Asterisks above and between the means in Figure 3a or over the bar in Figure 3b indicate that the means are significantly different between the treatments (one-way ANOVA, LSD test, * indicates *p* < 0.05, ** indicates *p* < 0.01).

Similar to previous results in pecan [19], the “food spray” results indicate that fermented molasses is an effective attractant for increasing ladybeetles and lacewings in the tree canopy (Figure 3a) and that these increases were associated with decreases in the abundance three pecan aphid species (Figure 3b). The increase in predators did not persist in the second week after treatment and resulted in a slight reduction in blackmargined aphids after the first week and moderate reductions in yellow and black pecan aphids two weeks after treatment.

## 3. Experimental Section

### 3.1. Seasonal Occurrence of Aphids and Aphidophagous Insects

A 7 ha block of a pecan orchard of “Desirable” variety trees, planted in 1986 at a spacing of 18 m × 18 m at a density of 60 trees per ha in Tift Co., GA, USA. (Coordinates 31.50922282331403, −83.6379396915435) was the site of the monitoring experiment. Trees were managed from 1986–2011 by following the recommendations of the University of Georgia Cooperative Extension Service. During the monitoring trials the trees were treated with fertilizer, herbicides and fungicides and not insecticides or miticides. The orchard floor was managed with herbicide-treated strips down the tree rows and mowed sod between the tree rows. The seasonal occurrence of the foliage-feeding aphids and associated aphidophagous insects were monitored in the orchard from 2006 to 2010 one-time per week from full leaf expansion in May to the onset of leaf fall in October each season. The aphids and aphidophagous insects were counted directly from samples of the pecan foliage. The sample unit was the leaves on one shoot or the current years stem growth. Shoots typical had 4–7 compound leaves (average 5.3 leaves per shoot) and three shoots per tree were collected per week from 8 trees at a height of ~4 m on the periphery of the tree canopy with a hand operated pole pruner and examined in the field for aphids and aphidophagous insects. Aphids were counted directly by counting the number of aphids in each of the five categories that could be distinguished with the naked eye. These were: yellow pecan aphid adults (YPA); blackmargined aphid adults (BMA); “so-called yellow aphid nymphs” (YAN) where nymphs of the yellow pecan aphid and blackmargined aphid were counted as one category; black pecan aphid adults (BPA); and, black pecan aphid nymphs (BPN). Yellow pecan and blackmargined aphids have similar reproductive rates [8,17] and we assumed that the ratio of yellow pecan aphid to blackmargined aphid adults (R_YPA:BMA_) was similar to the ratio of yellow pecan aphid to blackmargined aphid nymphs, and we calculated the total number of yellow pecan aphids and blackmargined aphids as:

(1) of yellow pecan aphids = # YPA + R_YPA:BMA_ × #YAN.
(2) of blackmargined aphids = # BMA + R_BMA:YPA_ × #YAN.
(3) black pecan aphids = # BPA + # BPN.

All life stages of the aphidophagous insects were counted on the samples and categorized in the field as ladybeetles, green lacewings, and parasitized aphid mummies. Aphidophagous insects were also sampled with malaise traps in 2010 and sticky boards in 2010 and 2011. During 2010, flying insects were collected with two malaise traps placed at the ground level between the tree rows to determine the flight periods of adult dolichopodid and syrphid flies. The traps caught many types of insects and the aphidophagous insects were sorted and counted from the trap catch. The malaise trap catch was analyzed by calculating the average for the two traps on each sampling date. Data from the weekly sampling with shoot samples and sticky boards was analyzed by calculating the mean and standard error for each insect category. During 2010 and 2011, aphidophagous insects were counted on 16 sticky boards placed in the tree canopy at a height of ~4–5 m and 16 sticky boards placed at the ground level on metal rebar stands at a height of 1.3 m. The sticky boards were painted with yellow satin finish latex paint (“Yellow Mustard” color code 3007-1A, manufactured by The Valspar Corp., Minneapolis, MN, USA) and coated with Tangle-Trap^®^ Sticky Coating (The Tanglefoot Co., Grand Rapids, MI, USA) on both sides of the boards in April and again in August during each season. During 2010 traps were 30 × 60 cm and hung with the 30 cm side parallel to the ground. Trap catch in the trees was very poor in 2010 and the trap size was increased it 60 × 60 cm in 2011. Trap catch data were analyzed each sampling date using one-way analysis of variance using PopTools Statistical Software [23] and LSD (*p* < 0.05) was calculated from the mean square error to determine if the means were significantly different.

### 3.2. Effect of “Food Spray” on Abundance of Aphids and Aphidophagous Insects

A trial to determine if a “food spray” would attract aphid predators into the tree canopy was conducted during the mid-summer blackmargined aphid outbreak of 2006. An aqueous solution of molasses (Grandma Food Products Ltd., Saint John, NB, Canada) (10 mL/L) and baker’s yeast (ACH Food Co., Memphis, TN, USA) (1 g/L) was applied to the foliage. The molasses concentration was 25% higher and the baker’s yeast concentration was 200% higher than in the treatment used for previous work on pecan [15]. The mixture was prepared on 9 July 2006 and allowed to ferment for one day at 25 C before the applications on 10 July 2006. Two 12-tree rows were selected for the trial separated by a buffer row of non-treated trees. Each 12 tree row was divided into 4 plots. Each plot had 3 adjacent trees planted in a row. The two treatments were randomly assigned to the 8 plots in a completely randomized design with 4 replications. The trees were monitored for aphids and aphidophagous insects one time per week for four weeks after the treatment date. Three shoots were sampled with a pruning pole from the canopy of the center tree in each replication from the mid-height (3–4 m) and insects were counted and categorized as yellow pecan, blackmargined or black pecan aphids, ladybeetles, lacewing, parasitized aphids or spiders. Means of the insect counts in each category were analyzed by one-way analysis of variance and a least significant difference was calculated to determine if the treatment means were significantly different.

## 4. Conclusions

Pecan aphid populations typically have two peaks of high abundance in the southeastern U.S. in the spring and fall with a hiatus during the mid-summer. The spring outbreaks are mixed populations of yellow pecan and blackmargined aphids and periods of high abundance occur after rapid exponential increases in the two populations followed by a brief (1–2 w) period of high abundance that is followed by a rapid decline in the mid-summer. Black pecan aphids typically occur as outbreaks in the late summer and fall. The yellow and blackmargined aphids were monitored on shoots with an average of 5.3 leaves per shoot. The action threshold for combined count of these two aphid species is 20 aphids per leaf or 106 aphids per shoot. Our results found that the combined counts of the two aphid species during the 6 seasons of this study exceeded the action threshold only one-time and that was during the mid-summer peak of 2006. Black pecan aphids on the other hand, exceeded the action threshold of 1 aphid per leaf or 5.3 aphids/shoot in 2006, 2009 and 2010. The peaks of black pecan aphids in the fall of 2011 exceeded the action threshold but only after the leaves were in senescence. The introduced ladybeetle, *H. axyridis* [24] is similar in its arboreal habit to the native ladybeetle, *Olla v-nigrum.* The results of the “food spray” experiment offer another tool for reduction of aphid abundance by managing aphid predators on organic pecan farms. Overall, the results are encouraging for managers of urban and organic pecan plantings in that aphids in the nontreated trees rarely developed yellow pecan and blackmargined aphid populations above the prescribed “action threshold” and the more injurious black pecan aphids were significantly reduced by the “food spray” treatment.
